# Perspectives in the management of thyroid orbitopathy

**DOI:** 10.1186/1756-6614-6-S2-A14

**Published:** 2013-04-05

**Authors:** Jacek Daroszewski

**Affiliations:** 1 Department of Endocrinology, Diabetes and Isotope Therapy, Medical University, Wroclaw, Poland

## 

Despite the huge progress in the understanding of the pathomechanism of thyroid orbitopathy (OT) done during recent years this extrathyroidal manifestation of Graves’ disease still remains one of the most complex problems of clinical endocrinology. The outcome of the management is not satisfying neither for patient nor for physicians. Some likelihood of the improvement of the results may be expected from innovative treatment options basing on the progress in understanding of mechanisms of autoimmunity and inflammation. On the other hand an effort must be done for the progress in the existing treatment regimes.

Glucocorticoids have been used in the treatment of severe OT for over 50 years but irrespectively of therapeutic regime any improvement in eye changes may be achieved only in ca. 70% of patients. Numerous schemes of glucocorticoid use should be validated, optimized and meet an individual approach as well as adhere to the local capabilities.

The common adjuvant treatment with selenium in Graves’ disease patients may prevent from a development of eye symptoms and possibly will improve the quality of life.

The mode of management of hyperthyroidism has an influence on OT. Medical and surgical therapy seem not to deteriorate the course of OT while radioiodine therapy may worsen eye symptoms. OT patients at high risk should get the preventive glucocorticoid treatment.

The access to a surgical treatment procedures as orbital decompression and rehabilitative surgery must be assured for OT patients and techniques of surgical procedures should be developed.

The results of anticytokine therapy are promising and the progress in this field may be expected while the value of newly developed TSH receptor blocking molecules needs further observations.

The frequency of circulating CD40(+) fibrocytes is markedly increased in patients with OT, suggesting that this receptor might represent a therapeutic target for OT.

An increase in a local steroidogenesis documented within orbital tissues in an active phase of OT rises the idea of the use of 11β hydroxysteroid dehydrogenase-1 inhibitors in OT patients. These drugs are under intensive clinical investigation for the treatment of type 2 diabetics.

Since recommendations basing on EBM according to the pivotal problems of the management OT are still lacking, the system of care for OT patients should be based on centres of excellence with proper experience and multidisciplinary approach.

